# Changes in Lipoinflammation Markers in People with Obesity after a Concurrent Training Program: A Comparison between Men and Women

**DOI:** 10.3390/ijerph17176168

**Published:** 2020-08-25

**Authors:** José Antonio González-Jurado, Walter Suárez-Carmona, Sergio López, Antonio Jesús Sánchez-Oliver

**Affiliations:** 1 Facultad de Ciencias del Deporte; Universidad Pablo de Olavide de Sevilla, 41013 Sevilla, Spain; jagonjur@upo.es; 2 Investigador Sistema de Información Científica de Andalucía, Universidad Pablo Olavide de Sevilla, 41013 Sevilla, Spain; waltersport10@gmail.com; 3 Laboratory of Cellular and Molecular Nutrition, Inst. de la Grasa, CSIC, 41013 Seville, Spain; serglom@us.es; 4 Department of Cell Biology, University of Seville, 41012 Seville, Spain; 5 Departamento de Motricidad Humana y Rendimiento Deportivo, Universidad de Sevilla, 41013 Sevilla, Spain

**Keywords:** overweight, sedentary lifestyle, physical exercise, adiponectin, leptin, adiponectin-leptin ratio

## Abstract

Obesity is related to low-grade systemic inflammation. This state of inflammation is characterized by the alteration in adipokine regulation, which may lead to a situation of cardiometabolic risk. The aim of this study was to evaluate the effects of a concurrent training program on markers of lipoinflammation in adult people with obesity, comparing the response to the training between men and women. A quasi-experimental, quantitative, and longitudinal study with a pre–post intervention was conducted. An 8-week concurrent training program was carried out, in which 26 individuals with obesity participated (mean ± SD; age = 46.38 ± 4.66) (BMI = 36.05 ± 4.99) (12 men and 14 women). Before and after the intervention period, blood samples were taken by percutaneous puncture. The blood levels of adiponectin and leptin were evaluated. Significant differences were obtained in the adiponectin–leptin ratio (A/L ratio) of the entire sample (*p* = 0.009, ES = 0.53), which indicates a decrease in the risk of cardiovascular diseases and lipoinflammation. There were no significant differences in the improvements observed after the training in A/L ratio between women (A/L change = +63.5%) and men (A/L change= +59.2%). It can be concluded that the combination of aerobic exercise and resistance training induced an improvement in markers of lipoinflammation and cardiometabolic risk in the individuals with obesity evaluated in this study.

## 1. Introduction

The worldwide expansion of obesity is a reason to consider it a pandemic nowadays. The World Health Organization (WHO) defines overweight and obesity as an abnormal or excessive accumulation of fat that poses a health risk [1]. Despite this relatively simplistic definition, obesity is a chronic, multifactorial, and multicausal disease, which corresponds to an alteration of the correct function of the adipose tissue in its capacity to store fat, both quantitatively and qualitatively [2]. In addition, it leads to an inflammatory condition of the aforementioned tissue (lipoinflammation), closely linked to metabolic disorders, which in turn are strongly associated with the metabolic syndrome [2].

Although an increase of the total body fat is associated with an increase of health risk, the amount of abdominal fat, particularly when it is found within the abdominal cavity, has been associated with an increase of the risk of comorbidity and mortality for different reasons. Thus, we observe a higher prevalence of type 2 diabetes, heart disease, stroke, sleep apnea, hypertension, dyslipidemia, insulin resistance, inflammation, and some types of cancer [2]. All these diseases have negative effects on the quality of life, work productivity, and the costs of medical assistance [3,4].

Lifestyle changes are a critical part of obesity prevention and intervention and are being pursued by public health organizations to promote health [5]. Physical inactivity and sedentary behavior are two powerful drivers of the obesity pandemic [6] and have been linked to an increased prevalence of disease and an increased risk of all-cause mortality [7].

Obesity has been recognized as a systemic low-grade inflammatory state, characterized by an increase of inflammatory factors and infiltration of immune cells, with no lesions or alterations in tissue structure or function [2]. It has been reported that obesity is associated with an atypical activation of proinflammatory signaling in the hypothalamus [8]. Although there are no alterations in cell morphology at the histological level, at the plasmatic level there is an increase of proinflammatory cytokines, such as TNF-α, IL-1b, IL-6, and C-reactive protein, as well as infiltration of macrophages and T lymphocytes in insulin-dependent tissues, among others, which triggers factors such as oxidative stress and insulin resistance [9].

Adipose tissue (AT) is a primordial connective tissue in the energy storage of every organism, and it mainly consists of adipocytes. It carries out autocrine, endocrine, and paracrine functions on the rest of the organs, participating in the regulation of energetic homeostasis, glucose and lipid metabolism, and hormone modulation, and it also plays a relevant role in the inflammatory process [10]. When a hypertrophic adipocyte exceeds its threshold capacity, its normal activity stops, showing a decrease in insulin sensitivity, hypoxia, an increase in cell stress, and consequently cell apoptosis, thus activating the inflammatory process [11]. AT secretes a series of proinflammatory and anti-inflammatory adipokines, of which the most popular are leptin and adiponectin. Leptin is a protein that performs its physiological activity through its receptor LEP-R (leptin receptor). The binding of leptin to its receptor in the hypothalamus triggers a series of chemical signals that affects the feeling of hunger and help to produce the feeling of satiety. An excess of leptin in the adipocyte promotes the inflammatory effect and decreases insulin sensitivity [12]. Adiponectin is the most abundant adipokine in human blood, with physiological levels of 5–30 ug/mL. It is secreted by the AT, mainly by white adipose tissue (WAT) [13]. Adiponectin is a protein hormone that plays an anti-inflammatory role within the adipose tissue. Previous studies have shown insulin-sensitizing, anti-atherogenic, and anti-inflammatory effects [2]. This role of adiponectin in the inflammatory process is particularly positive in patients with metabolic diseases such as type 2 diabetes mellitus and obesity, who have shown low-grade chronic inflammation and low serum adiponectin concentrations [4]. Therefore, leptin and adiponectin play an important role in the health of the individual and his adipose tissue. Leptin regulates innate and adaptive responses through the modulation of the activity and proliferation of immune cells; thus, it can regulate the polarization toward a proinflammatory phenotype. On the other hand, adiponectin shows anti-inflammatory properties, stimulates lipid oxidation, and improves glucose metabolism by increasing insulin sensitivity. Adipocyte hypertrophy produces an imbalance in these hormones, promoting the production of leptin and reducing that of adiponectin; participating in adverse signals from adipose tissue that can lead to secondary tissue damage that increases concomitant lipotoxicity [2,14]. Therefore, the adiponectin/leptin ratio (A/L ratio) is identified as a marker of adipose tissue failure. It has been demonstrated that this index is strongly associated with insulin resistance, showing a stronger relationship with the risk of developing type 2 diabetes compared to the analysis of leptin or adiponectin separately [15].

The A/L ratio is a good indicator of a malfunctioning adipose tissue and it is negatively associated with markers of low-grade chronic inflammation; thus, it can be a useful stimulator of the cardiometabolic risk associated with obesity [16]. Although the measurement method used must be taken into account, it is considered that a normal A/L ratio should have values above 1.0 (adiponectin levels expressed in μg/mL and leptin levels in ng/mL), whereas an A/L ratio between 0.5 and 1.0 represents a moderate/medium cardiometabolic risk, and a proportion below 0.5 is a serious indicator of cardiometabolic risk [17].

Numerous authors agree in the effects of physical exercise on the AT [18,19,20,21], such as changes in the AT phenotype that lead to a significant decrease of M1 macrophages and an increase of M2 macrophages, as well as the secretion of more anti-inflammatory cytokines, such as IL-6 and TNF-α [22]. Other researchers have studied the possible changes in the signaling pathways after implementing exercise programs, reporting a decrease of postprandial lipemia, a reduction in NF-kB signaling, and an increase in AMPK signaling (showing anti-inflammatory properties) [23,24,25]. The interest of these studies is increasingly focused on determining the most adequate training to reduce the inflammatory state and its effects on metabolic syndrome in people with obesity. There is scientific evidence that aerobic training improves the levels of proinflammatory cytokines (TNF-α, IL-6, and IL-8) in people with obesity and type 2 diabetes [26]. In fact, aerobic or cardiorespiratory exercise programs are the most frequently implemented [27]. However, other studies have analyzed the impact of resistance training or a combination of resistance and aerobic training on low-grade chronic inflammation in people with obesity and overweight [28,29,30,31]. Even methodologies of highly specialized training with high-intensity interval training (HIIT) have been implemented to compare their effect on the inflammatory state of people with obesity and normal weight [32].

The aim of this study was to evaluate the effects of an 8-week concurrent training program on markers of lipoinflammation (adiponectin, leptin, and adiponectin-leptin ratio) in 26 adult people with obesity, comparing the response to the training between men and women.

## 2. Materials and Methods

**Figure 1:**
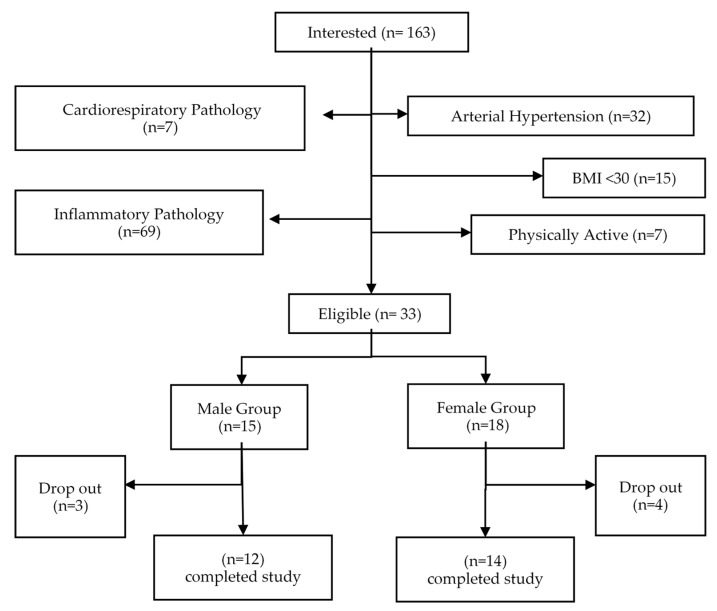
Recruitment flowchart.

This is a quasi-experimental, quantitative, and longitudinal study with a pre–post intervention. A total of 26 individuals participated in the study (12 men and 14 women), with an age range of 38 to 54 years (46.38 ± 4.66) and obesity (BMI = 36.05 ± 4.99). Table 1 shows baseline and Figure 1 shows a flowchart of the participant selection process followed in the study.

The following inclusion criteria were applied; (i) 35–55 years of age; (ii) BMI: > 30; (iii) abdominal perimeter: >90 cm in women and >100 cm in men; (iv) fat%: >40 in women and >30 in men; (v) signed informed consent; and (vi) official medical authorization that allowed the participant to carry out the program of planned exercises. Similarly, the following exclusion criteria were applied; (i) diagnosis of a pathology involving an inflammatory process (e.g., myocardial infarction in the last 12 months, rheumatoid arthritis, fibromyalgia, and arthrosis), and (ii) failing to complete 75% of the training sessions. The study meets the requirements of the Declaration of Helsinki of the World Medical Association (2013) about the ethical principles for research with human beings, and it was approved by the Ethics Committee of the University of Pablo de Olavide (Seville, Spain).

**Figure 2:**
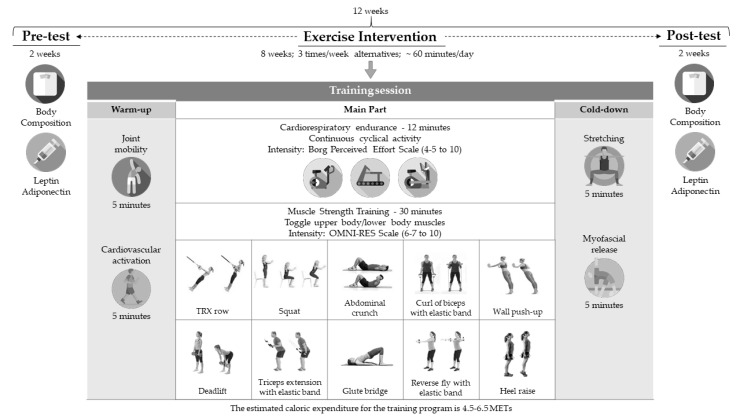
Summary of the training program and training session.

The independent variable was a concurrent training program carried out for 8 weeks, with 3 weekly sessions of ~60 min each in alternate days (Monday, Wednesday, and Friday). The trainings were conducted in the time frame of 5:30 p.m. to 8:30 p.m. to accommodate the individual needs of each participant (work, family, personal, etc.) (Figure 2).

Each training session began with a general joint mobility (5 min) and cardiovascular activation (5 min), followed by a cardiorespiratory resistance exercise (three possible options: treadmill, cycle ergometer, or elliptical bike). This task was carried out for 12 min at a medium–low intensity. The intensity was determined using Borg’s rate of perceived exertion (RPE), aiming to reach a RPE value of 4–5 out of 10 [33]. Then, a resistance training was conducted, with a 10-station circuit. Figure 2 shows the ten exercises proposed, which involve the main muscle groups used in the most habitual basic motor skills and global motor actions of daily living (walking, running, throwing, carrying weight, etc.).

The working time in each station was 45 s in the first two weeks, and it was increased to 60 s from the third week. The resting time between exercises was 45 s in the first two week and 30 s from the second week. In the first two weeks, two circuits were completed in each session, with a passive rest of 2 min. After the third week, the circuit was completed 3 times in each session, keeping the resting time to 2 min. The intensity of the exertion was measured through the RPE scale OMNI-RES, aiming to reach and maintain values of 6–7 out of 10 [34]. Every session ended with cooling exercises (Figure 2).

The planned training (temporalization, organization, methodology, training load levels, characteristics of the exercises, etc.) allowed each participant to undergo an individualized training stimulus. In this study, it was never intended to standardize a closed and rigid training program. In this sense, each participant, depending on their physical capacity, underwent a training stimulus that posed a relative subjective intensity similar to that of the rest of the sample. Therefore, simple exercises were selected, in terms of execution and adaptation to the individual capacities.

The blood markers analyzed were leptin and adiponectin. Endovenous puncture equipment and tubes were used for the collection of biological samples. Endovenous blood was extracted by percutaneous puncture using appropriate needles and syringes. These techniques are minimally invasive, practically harmless, and pose no serious threat to the participants.

For the statistical analyses, the IBM SPSS Statistics 23 software (SPSS Inc., Chicago, IL, USA) was used. Regarding the descriptive statistics, the mean and standard deviation were calculated. The reliability of the measurements was estimated at a 95% confidence interval for the mean. Regarding the inferential statistics, a paired-sample t or Wilcoxon test was conducted for intragroup comparisons, depending on the normality (assessed through the Shapiro–Wilk test), and an independent-samples t or Mann–Whitney U test, depending on the normality and the Levene test. Cohen’s d effect size was also calculated [35], considering values of d < 0.3 as small, d = 0.3–0.5 as moderate, d = 0.5–0.7 as large, d = 0.7–0.9 as very large, and d > 0.9 as extremely large [36].

## 3. Results

Table 2 shows the pre–post comparison for the entire sample. As can be seen, there was a significant increase in the adiponectin/leptin ratio at the end of the intervention period with respect to the baseline situation, with a moderate effect size. There were no significant differences in the results obtained in the variables “adiponectin” and “leptin”.

Table 3 shows the results obtained when comparing the variables “adiponectin” and “leptin” before and after the intervention in the women’s group. There were no significant differences in the levels of adipokines. However, the A/L ratio did show a statistically significant improvement (*p* = 0.05) with a large effect size (d = 0.55).

Table 4 shows the values obtained in the men’s group, which were similar to those observed in the women’s group, i.e., there were no significant differences in the levels of adipokines between the pre-test and the post-test. In this case, although the A/L ratio improved, this increase was not statistically significant (*p* = 0.08); however, the effect size of this increase can be considered as moderate (d = 0.46).

**Figure 3:**
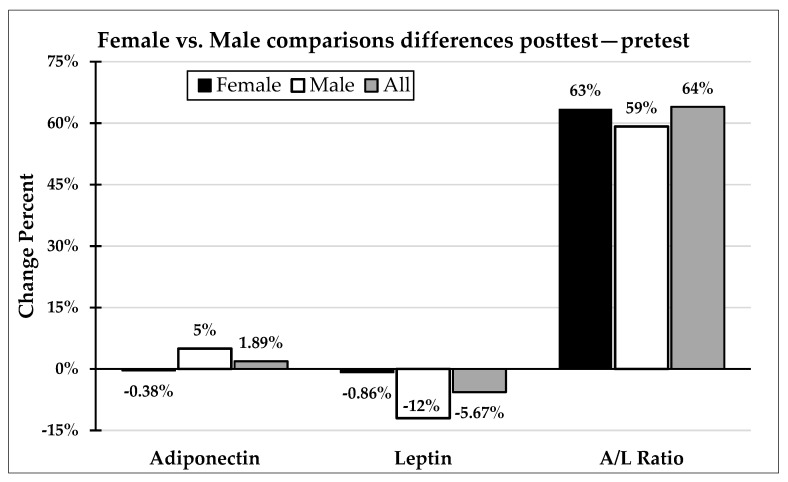
Changes after intervention comparisons. No significant differences were found between sex groups. Student *t*-test or Mann–Whitney U were carried out depending on the normality and Levene test.

The results of the intergroup comparisons (women vs. men) are represented in Figure 3. The pre–post differences are compared, expressed in percentages. As can be observed, the differences between men and women are very small; in fact, there were no statistically significant differences in any of the three variables. It is worth highlighting that the A/L ratio increased considerably in the two groups and in a similar manner, suggesting that both improved this index to the same extent.

## 4. Discussion

The aim of this study was to analyze the effects of a physical exercise program based on concurrent training on lipoinflammation marker adipokines in adults with obesity, comparing the responses between men and women.

Table 2 compares the results of the entire sample (n = 26), showing that the initial average leptin levels were 44.09 ng/mL. These levels are associated with greater insulin resistance, and they are usually found in people with a BMI above 30. The results show an improvement of these values after the intervention, from 44.09 down to 41.59 ng/mL, although this decrease was small (−2.5 ng/mL) and not statistically significant. The changes in adiponectin were also positive, that is, there was an increase in its blood levels (+0.32 ug/mL), although this increase was not statistically significant either.

The results are presented separately by sex in Table 3 and Table 4. These results are similar to those obtained considering the entire sample. There were signs of improvement in adiponectin and leptin, although such changes were very small. On the other hand, there were more considerable differences in the increases in the A/L ratio, which were significant in the women’s group (*p* = 0.5) and close to statistical significance in the men’s group (*p* = 0.08), with a large effect size in women (0.55) and moderate in men (0.46).

In numerous studies, people with obesity have conducted different training programs, reporting improvements in the plasmatic levels of leptin [37,38,39,40], with significant decreases. Likewise, a large number of studies have analyzed the effects of different physical exercise programs on the concentrations of adiponectin [41,42,43,44].

In the present study, the changes in adiponectin and leptin were lower, probably due to the fact that the intervention period was considerably shorter compared to that of the cited studies [37,42,45], or because their interventions evaluated the acute effect of exercise programs of higher intensity [43,44]. Other studies combined the training with the administration of ergogenic or anti-inflammatory supplements [41].

The improvements in the levels of adiponectin and leptin, analyzed independently, were not significant; however, the A/L ratio did obtain a significant increase after the training period (*p* = 0.009), with a large effect size (0.53). The A/L ratio is a biomarker that has been well studied in its relationship with lipoinflammation [16], and as an indicator of obesity and metabolic risk [46,47,48]. However, only few studies have analyzed the effect of planned and controlled physical exercise programs on this marker (A/L ratio) in people with obesity, and most of these studies evaluated these two adipocytokines independently [27,30,49,50]. All of them report significant improvements after exercise, both an increase in adiponectin and a decrease in leptin, which is in line with the results of the present study. A novelty of our study is that it analyses the effect of a concurrent or combined training program on a specific lipoinflammation marker (A/L ratio), obtaining significant improvements after 8 weeks of intervention. One of the factors in controversy or disagreement with the scientific literature is related to the characteristics of the training programs implemented. In this sense, most of the studies that report positive results in the levels of both adipokines (adiponectin and leptin) after the exercise program, carried out aerobic or cardiorespiratory trainings [49,51,52,53]. Few studies have included resistance training [30,31], although concurrent or combined training programs are being applied increasingly frequently, as they seem to be more effective, as was demonstrated in the current study.

Another finding among the obtained results is related to the response of the lipoinflammation markers analyzed based on sex. Most studies have been conducted with samples of a single gender. In a meta-analysis published in 2017, in which 28 RCTs were included, only two of these recruited both men and women in their samples [27]. One of these studies only evaluated the effects of exercise on leptin and the participants were adolescents [54], and in another study the participants were children and the authors only evaluated adiponectin [55]. Another meta-analysis published in 2018 included 22 trials with 2996 individuals. These authors concluded aerobic exercise increased adiponectin and reduced leptin levels in prediabetic and diabetic adults. Most trials included both sexes, but there were not compared men versus women. In addition, the studies, the population analyzed, and the design of physical exercise interventions heterogeneity was found [56]. Figure 3 compares the change percentages in the three variables analyzed between men and women, clearly showing that sex was not a determining factor in the response of these adipokines to exercise, obtaining no significant differences between men and women in the response to the training program implemented.

A current narrative review about the effects of physical activity (PA) on adipokine levels in individuals with overweight and obesity has analyzed approximately 90 different investigations on adipokine levels in individuals with overweight/obesity were reviewed. The results support the benefits of exercise regardless of the mode (resistance vs. aerobic), intensity, and cohort (healthy vs. diabetes) on adipokines levels. However, several confounding factors (frequency, intensity, time, and type of exercise) can alter the magnitude of these effects [57].

A low number of experimental subjects and the lack of a control group are the two major limitations of this study. Despite this, the present study has been able to demonstrate that a simple and a short program of physical exercise improves the markers of lipoinflammation studied and, therefore, the inflammatory health of the participants. However, cautious interpretation of current findings is warranted.

There is a large number of studies about the effect of exercise on low-grade chronic inflammation; however, there is also a wide methodological variety, diversity in the characteristics of the participants, and multiple physical exercise programs implemented with important differences in the characteristics of the training loads. Therefore, it seems obvious that further research is still required in this field.

## 5. Conclusions

The participants of this study started with a low A/L ratio, with risk of low-grade chronic inflammation. Both women and men improved in the three indicators analyzed, although statistically significant improvements with a large effect size in women and moderate in men were obtained in A/L ratio, with these improvements being more evident when considering the entire sample without sex differentiation. In fact, when comparing women and men, no difference was found in the response of the adipokines to the exercise program based on sex.

## Figures and Tables

**Table 1:** Participants’ baselines.

	Male		Female		All	
	Mean ± SD	Range	Mean ± SD	Range	Mean ± SD	Range
Age (yr)	46.42 ± 5	38–54	46.36 ± 4.53	40–53	46.38 ± 4.66	38–54
Height (m)	1.75 ± 0.07	1.6–1.9	1.61 ± 0.08	1.44–1.73	1.67 ± 0.10	1.44–1.9
Weight (kg)	109.9 ± 16.35	87–140	94.67 ± 21.95	70.7–147	101.73 ± 20.7	70.7–147
BMI	35.98 ± 3.98	30.1–42.2	36.12 ± 5.87	30.05–49.69	36.05 ± 4.99	30–49.7
BFM (kg)	43.86 ± 11.08	24–58.4	45.11 ± 14.70	30.8–79.3	44.53 ± 12.92	24–79.3
BFMP (%)	39.53 ± 5.94	28–48.8	47.59 ± 4.63	39–53.9	43.87 ± 6.59	28–53.9

BMI: Body Mass Index. BFM: Body Fat Mass; BFMP: Body Fat Mass Percent.

**Table 2:** Intragroup comparisons. Whole sample (n = 26).

	Pre-Test		Post-Test		Change		*p*^§^ Value	E.S ^¥^
	Mean ± SD	CI (95%)	Mean ± SD	CI (95%)	Mean ± SD	CI (95%)		
Adiponecti (ug/mL)	16.94 ± 4.89	(14.9–18.6)	17.3 ± 5.1	(15.2–19.3)	0.32 ± 3.04	(−0.91–1.55)	0.20	0.06
Leptin (ng/mL)	44.09 ± 24.3	(34.3–53.9)	41.6 ± 30.5	(29.3–53.9)	−2.5 ± 20.1	(−10.6–5.59)	0.47	−0.09
A/L ratio *	0.5 ± 0.32	(0.37–0.63)	0.82 ± 0.79	(0.5–1.14)	0.32 ± 0.57	(0.09–0.55)	0.009 *	0.53

^§^ Student *t*-test or Wilcoxon depending on the normality; ^¥^ Cohen’s effect size (d < 0.3 small; d = 0.3–0.5 moderate; d = 0.5–0.7 large; d = 0.7–0.9 very large and d > 0.9 extremely large). * Adiponectin/leptin ratio.

**Table 3:** Intragroup comparisons. Women (N = 14).

	Pre-Test		Post-Test		Change		*p*^§^ Value	E.S ^¥^ CI (95%)
	Mean ± SD	CI (95%)	Mean ± SD	CI (95%)	Mean ± SD	Mean ± SD		
Adiponectin (ug/mL)	18.24 ± 3.8	(16.04–20.43)	18.17 ± 1.16	(15.67–20.67)	−0.06 ± 3.63	(−2.16–2.03)	0.55	−0.02
Leptin (ng/mL)	46.5 ± 23.8	(32.7–60.2)	46.1 ± 37.6	(2.44–6.78)	−0.4 ± 21.52	(−12.8–12.0)	0.82	−0.01
A/L ratio *	0.51 ± 0.28	(0.35–0.67)	0.85 ± 0.8	(0.39–1.31)	0.34 ± 0.62	(−0.01–0.7)	0.05*	0.55

^§^ Student *t*-test or Wilcoxon depending on the normality. ^¥^ Cohen’s effect size (d < 0.3 small; d = 0.3–0.5 moderate; d = 0.5–0.7 large; d = 0.7–0.9 very large and d > 0.9 extremely large). * Adiponectin/leptin ratio.

**Table 4:** Intragroup comparisons. Men (N = 12).

	Pre-Test		Post-Test		Change		*p* Value ^§^	E.S ^¥^
	Mean ± SD	CI (95%)	Mean ± SD	CI (95%)	Mean ± SD	CI (95%)		
Adiponectin (ug/mL)	15.43 ± 5.71	(11.8–19.1)	16.2 ± 5.71	(12.57–19.83)	0.76 ± 2.25	(−0.67–2.2)	0.26	0.12
Leptin (ng/mL)	41.32 ± 25.6	(25.1–57.6)	36.36 ± 19.86	(23.7–48.9)	−4.96 ± 18.82	(−16.9–6.9)	0.38	−0.22
A/L ratio *	0.49 ± 0.37	(0.25–0.73)	0.78 ± 0.81	(0.27–1.29)	0.29 ± 0.53	(−0.05–0.63)	0.08	0.46

^§^ Student *t*-test or Wilcoxon according to normality. ^¥^ Cohen’s effect size (d < 0.3 small; d = 0.3–0.5 moderate; d = 0.5–0.7 large; d = 0.7–0.9 very large and d > 0.9 extremely large). * Adiponectin/Leptin ratio.

## References

[1] W.H.O. World Health Organization Obesity and Overweight Fact Sheet. https://www.who.int/news-room/fact-sheets/detail/obesity-and-overweight.

[2] Suárez-Carmona W., Sánchez-Oliver A.J., González-Jurado J.A. (2017). Pathophysiology of obesity: Current view. Rev. Chil. Nutr..

[3] Sánchez-Oliver A.J., Martín-García C., Gálvez-Ruiz P., González-Jurado J.A. (2018). Mortality and Economic Expenses of Cardiovascular Diseases Caused by Physical Inactivity in Spain. J. Phys. Educ. Sport.

[4] Choi H.M., Doss H.M., Kim K.S. (2020). Multifaceted physiological roles of adiponectin in inflammation and diseases. Int. J. Mol. Sci..

[5] Galán-Lopez P., Sánchez-Oliver A.J., Pihu M., Gísladóttír T., Domínguez R., Ries F. (2020). Association between Adherence to the Mediterranean Diet and Physical Fitness with Body Composition Parameters in 1717 European Adolescents: The AdolesHealth Study. Nutrients.

[6] Swinburn B.A., Sacks G., Hall K.D., McPherson K., Finegood D.T., Moodie M.L., Gortmaker S.L. (2011). The global obesity pandemic: Shaped by global drivers and local environments. Lancet.

[7] Young D.R., Hivert M.-F., Alhassan S., Camhi S.M., Ferguson J.F., Katzmarzyk P.T., Lewis C.E., Owen N., Perry C.K., Siddique J. (2016). Sedentary Behavior and Cardiovascular Morbidity and Mortality: A Science Advisory From the American Heart Association. Circulation.

[8] Cai D., Khor S. (2019). “Hypothalamic Microinflammation” Paradigm in Aging and Metabolic Diseases. Cell Metab..

[9] León-Pedroza J.I., González-Tapia L.A., del Olmo-Gil E., Castellanos-Rodríguez D., Escobedo G., González-Chávez A. (2015). Low-grade systemic inflammation and the development of metabolic diseases: From the molecular evidence to the clinical practice. Cirugía Cir..

[10] Izaola O., de Luis D., Sajoux I., Domingo J.C., Vidal M. (2015). Inflammation and obesity (Lipoinflammation). Nutr. Hosp..

[11] Klöting N., Blüher M.J.R.i.E., Disorders M. (2014). Adipocyte dysfunction, inflammation and metabolic syndrome. Rev. Endocr. Metab. Disord..

[12] Francisco V., Pino J., Campos-Cabaleiro V., Ruiz-Fernández C., Mera A., González-Gay M.A., Gómez R., Gualillo O. (2018). Obesity, fat mass and immune system: Role for leptin. Front. Physiol..

[13] Obata Y., Yamada Y., Takahi Y., Baden M.Y., Saisho K., Tamba S., Yamamoto K., Umeda M., Furubayashi A., Matsuzawa Y. (2013). Relationship between serum adiponectin levels and age in healthy subjects and patients with type 2 diabetes. Clin. Endocrinol..

[14] Kojta I., Chacińska M., Błachnio-Zabielska A. (2020). Obesity, Bioactive Lipids, and Adipose Tissue Inflammation in Insulin Resistance. Nutrients.

[15] Chou H.-H., Hsu L.-A., Wu S., Teng M.-S., Sun Y.-C., Ko Y.-L. (2014). Leptin-to-adiponectin ratio is related to low grade inflammation and insulin resistance independent of obesity in non-diabetic Taiwanese: A cross-sectional cohort study. Acta Cardiol. Sin..

[16] Frühbeck G., Catalán V., Rodríguez A., Ramírez B., Becerril S., Salvador J., Colina I., Gómez-Ambrosi J. (2019). Adiponectin-leptin ratio is a functional biomarker of adipose tissue inflammation. Nutrients.

[17] Frühbeck G., Catalán V., Rodríguez A., Gómez-Ambrosi J. (2018). Adiponectin-leptin ratio: A promising index to estimate adipose tissue dysfunction. Relation with obesity-associated cardiometabolic risk. Adipocyte.

[18] Soltani N., Marandi S.M., Kazemi M., Esmaeil N. (2020). The Exercise Training Modulatory Effects on the Obesity-Induced Immunometabolic Dysfunctions. Diabetes Metab. Syndr. Obes..

[19] Goh J., Goh K.P., Abbasi A. (2016). Exercise and Adipose Tissue Macrophages: New Frontiers in Obesity Research?. Front. Endocrinol..

[20] Bruun J.M., Helge J.W., Richelsen B., Stallknecht B. (2006). Diet and exercise reduce low-grade inflammation and macrophage infiltration in adipose tissue but not in skeletal muscle in severely obese subjects. Am. J. Physiol. Endocrinol. Metab..

[21] Silveira L.S., Antunes B.D.M.M., Minari A., Dos Santos R.V.T., Neto J.C.R., Lira F.S. (2016). Macrophage Polarization: Implications on Metabolic Diseases and the Role of Exercise. Crit. Rev. Eukaryot. Gene Expr..

[22] Dieli-Conwright C.M., Parmentier J.-H., Sami N., Lee K., Spicer D., Mack W.J., Sattler F., Mittelman S.D. (2018). Adipose tissue inflammation in breast cancer survivors: Effects of a 16-week combined aerobic and resistance exercise training intervention. Breast Cancer Res. Treat..

[23] Allen J., Sun Y., Woods J.A. (2015). Exercise and the Regulation of Inflammatory Responses. Prog. Mol. Biol. Transl. Sci..

[24] Woods J.A., Vieira V.J., Keylock K.T. (2009). Exercise, inflammation, and innate immunity. Immunol. Allergy Clin. N. Am..

[25] Lehnig A.C., Stanford K.I. (2018). Exercise-induced adaptations to white and brown adipose tissue. J. Exp. Biol..

[26] El-Kader S.M.A., Al-Jiffri O.H., Al-Shreef F.M. (2015). Aerobic exercises alleviate symptoms of fatigue related to inflammatory cytokines in obese patients with type 2 diabetes. Afr. Health Sci..

[27] Yu N., Ruan Y., Gao X., Sun J. (2017). Systematic Review and Meta-Analysis of Randomized, Controlled Trials on the Effect of Exercise on Serum Leptin and Adiponectin in Overweight and Obese Individuals. Horm. Metab. Res..

[28] Annibalini G., Lucertini F., Agostini D., Vallorani L., Gioacchini A., Barbieri E., Guescini M., Casadei L., Passalia A., Del Sal M. (2017). Concurrent aerobic and resistance training has anti-inflammatory effects and increases both plasma and leukocyte levels of IGF-1 in late middle-aged type 2 diabetic patients. Oxidative Med. Cell. Longev..

[29] Venojärvi M., Wasenius N., Manderoos S., Heinonen O.J., Hernelahti M., Lindholm H., Surakka J., Lindström J., Aunola S., Atalay M. (2013). Nordic walking decreased circulating chemerin and leptin concentrations in middle-aged men with impaired glucose regulation. Ann. Med..

[30] Winzer B.M., Paratz J.D., Whitehead J.P., Whiteman D.C., Reeves M.M. (2015). The feasibility of an exercise intervention in males at risk of oesophageal adenocarcinoma: A randomized controlled trial. PLoS ONE.

[31] Ligibel J.A., Giobbie-Hurder A., Olenczuk D., Campbell N., Salinardi T., Winer E.P., Mantzoros C.S. (2009). Impact of a mixed strength and endurance exercise intervention on levels of adiponectin, high molecular weight adiponectin and leptin in breast cancer survivors. Cancer Causes Control..

[32] Dorneles G.P., Haddad D.O., Fagundes V.O., Vargas B.K., Kloecker A., Romão P.R., Peres A. (2016). High intensity interval exercise decreases IL-8 and enhances the immunomodulatory cytokine interleukin-10 in lean and overweight–obese individuals. Cytokine.

[33] Borg E., Kaijser L. (2006). A comparison between three rating scales for perceived exertion and two different work tests. Scand. J. Med. Sci. Sports.

[34] Robertson R.J., Goss F.L., Rutkowski J., Lenz B., Dixon C., Timmer J., Frazee K., Dube J., Andreacci J. (2003). Concurrent validation of the OMNI perceived exertion scale for resistance exercise. Med. Sci. Sports Exerc..

[35] Cohen J. (1988). Statistical Power Analysis for the Behavioral Sciences.

[36] Hopkins W., Marshall S., Batterham A., Hanin J. (2009). Progressive statistics for studies in sports medicine and exercise science. Med. Sci. Sports Exerc..

[37] Tremblay A., Dutheil F., Drapeau V., Metz L., Lesour B., Chapier R., Pereira B., Verney J., Baker J.S., Vinet A. (2019). Long-term effects of high-intensity resistance and endurance exercise on plasma leptin and ghrelin in overweight individuals: The RESOLVE Study. Appl. Physiol. Nutr. Metab. Physiol. Appl. Nutr. Metab..

[38] de Souza D.C., Matos V.A.F., dos Santos V.O.A., Medeiros I.F., Marinho C.S.R., Nascimento P.R.P., Dorneles G.P., Peres A., Müller C.H., Krause M. (2018). Effects of high-intensity interval and moderate-intensity continuous exercise on inflammatory, leptin, IgA, and lipid peroxidation responses in obese males. Front. Physiol..

[39] Rostás I., Pótó L., Mátrai P., Hegyi P., Tenk J., Garami A., Illés A., Solymár M., Pétervári E., Szűcs Á. (2017). In middle-aged and old obese patients, training intervention reduces leptin level: A meta-analysis. PLoS ONE.

[40] Salvadori A., Fanari P., Brunani A., Marzullo P., Codecasa F., Tovaglieri I., Cornacchia M., Palmulli P., Longhini E. (2015). Leptin level lowers in proportion to the amount of aerobic work after four weeks of training in obesity. Horm. Metab. Res..

[41] Bagheri R., Rashidlamir A., Ashtary-Larky D., Wong A., Grubbs B., Motevalli M.S., Baker J.S., Laher I., Zouhal H. (2020). Effects of green tea extract supplementation and endurance training on irisin, pro-inflammatory cytokines, and adiponectin concentrations in overweight middle-aged men. Eur. J. Appl. Physiol..

[42] Kim D.Y., Seo B.D., Kim D.J. (2014). Effect of walking exercise on changes in cardiorespiratory fitness, metabolic syndrome markers, and high-molecular-weight adiponectin in obese middle-aged women. J. Phys. Ther. Sci..

[43] Saunders T.J., Palombella A., McGuire K.A., Janiszewski P.M., Després J.P., Ross R. (2012). Acute exercise increases adiponectin levels in abdominally obese men. J. Nutr. Metab..

[44] Kelly K.R., Blaszczak A., Haus J.M., Patrick-Melin A., Fealy C.E., Solomon T.P.J., Kalinski M.I., Kirwan J.P. (2012). A 7-d exercise program increases high-molecular weight adiponectin in obese adults. Med. Sci. Sports Exerc..

[45] Ibañez J., Izquierdo M., Martínez-Labari C., Ortega F., Grijalba A., Forga L., Idoate F., García-Unciti M., Fernández-Real J.M., Gorostiaga E.M. (2010). Resistance training improves cardiovascular risk factors in obese women despite a significative decrease in serum adiponectin levels. Obesity.

[46] de Piano-Ganen A., Masquio D.C.L., Dâmaso A.R., Oyama L.M., Estadella D., Chamas A., do Nascimento C.M.P.O. (2017). Serum myristic fatty acid negatively correlates with anti-inflammatory adiponectin/leptin ratio in obese adolescents: Effects of long-Term therapy. Mundo Saude.

[47] Musil F., Blaha V., Ticha A., Hyspler R., Haluzik M., Lesna J., Smahelova A., Sobotka L. (2015). Effects of body weight reduction on plasma leptin and adiponectin/leptin ratio in obese patients with type 1 diabetes mellitus. Physiol. Res..

[48] Mirza S., Qu H.Q., Li Q., Martinez P.J., Rentfro A.R., McCormick J.B., Fisher-Hoch S.P. (2011). Adiponectin/leptin ratio and metabolic syndrome in a mexican american population. Clin. Investig. Med..

[49] Kim Y.S., Nam J.S., Yeo D.W., Kim K.R., Suh S.H., Ahn C.W. (2015). The effects of aerobic exercise training on serum osteocalcin, adipocytokines and insulin resistance on obese young males. Clin. Endocrinol..

[50] Abbenhardt C., McTiernan A., Alfano C.M., Wener M.H., Campbell K.L., Duggan C., Foster-Schubert K.E., Kong A., Toriola A.T., Potter J.D. (2013). Effects of individual and combined dietary weight loss and exercise interventions in postmenopausal women on adiponectin and leptin levels. J. Intern. Med..

[51] Beavers K.M., Ambrosius W.T., Nicklas B.J., Rejeski W.J. (2013). Independent and combined effects of physical activity and weight loss on inflammatory biomarkers in overweight and obese older adults. J. Am. Geriatr. Soc..

[52] Auerbach P., Nordby P., Bendtsen L.Q., Mehlsen J.L., Basnet S.K., Vestergaard H., Ploug T., Stallknecht B. (2013). Differential effects of endurance training and weight loss on plasma adiponectin multimers and adipose tissue macrophages in younger, moderately overweight men. Am. J. Physiol. Regul. Integr. Comp. Physiol..

[53] Akbarpour M. (2013). The effect of aerobic training on serum adiponectin and leptin levels and inflammatory markers of coronary heart disease in obese men. Biol. Sport.

[54] Ackel-D’Elia C., Carnier J., Bueno C.R.J., Campos R.M., Sanches P.L., Clemente A.P., Tufik S., de Mello M.T., Dâmaso A.R. (2014). Effects of different physical exercises on leptin concentration in obese adolescents. Int. J. Sports Med..

[55] Murphy E.C., Carson L., Neal W., Baylis C., Donley D., Yeater R. (2009). Effects of an exercise intervention using Dance Dance Revolution on endothelial function and other risk factors in overweight children. Int. J. Pediatr. Obes..

[56] Becic T., Studenik C., Hoffmann G. (2018). Exercise Increases Adiponectin and Reduces Leptin Levels in Prediabetic and Diabetic Individuals: Systematic Review and Meta-Analysis of Randomized Controlled Trials. Med. Sci..

[57] Saeidi A., Haghighi M.M., Kolahdouzi S., Daraei A., Ben Abderrahmane A., Essop M.F., Laher I., Hackney A.C., Zouhal H. (2020). The effects of physical activity on adipokines in individuals with overweight/obesity across the lifespan: A narrative review. Obes Rev..

